# Excitation-Dependent High-Lying Excitonic Exchange *via* Interlayer Energy Transfer from *Lower*-*to*-*Higher* Bandgap 2D Material

**DOI:** 10.1021/acs.nanolett.3c01127

**Published:** 2023-06-08

**Authors:** Arka Karmakar, Tomasz Kazimierczuk, Igor Antoniazzi, Mateusz Raczyński, Suji Park, Houk Jang, Takashi Taniguchi, Kenji Watanabe, Adam Babiński, Abdullah Al-Mahboob, Maciej R. Molas

**Affiliations:** † Institute of Experimental Physics, Faculty of Physics, University of Warsaw, Pasteura 5, 02-093 Warsaw, Poland; ‡ Center for Functional Nanomaterials, Brookhaven National Laboratory, Upton, New York 11973, USA; § International Center for Materials Nanoarchitectonics, National Institute for Materials Science, 1-1 Namiki, Tsukuba, Ibaraki 305-0044, Japan; ∥ Research Center for Functional Materials, National Institute for Materials Science, 1-1 Namiki, Tsukuba, Ibaraki 305-0044, Japan

**Keywords:** 2D material, MoS_2_, WSe_2_, heterostructure, energy transfer, band-nesting

## Abstract

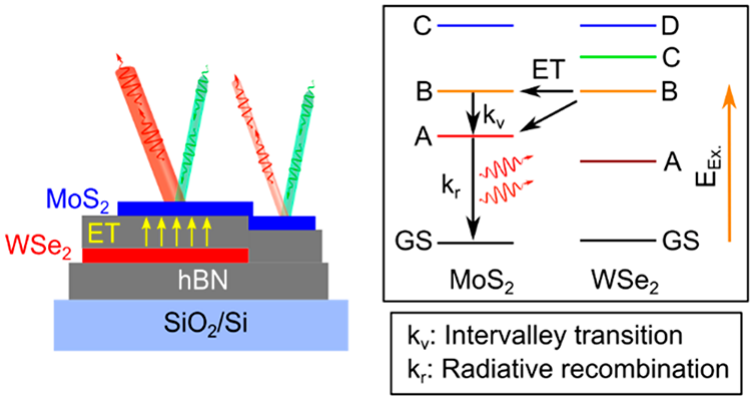

High light absorption (∼15%) and strong photoluminescence (PL) emission in monolayer (1L) transition metal dichalcogenides (TMDs) make them ideal candidates for optoelectronic device applications. Competing interlayer charge transfer (CT) and energy transfer (ET) processes control the photocarrier relaxation pathways in TMD heterostructures (HSs). In TMDs, long-distance ET can survive up to several tens of nm, unlike the CT process. Our experiment shows that an efficient ET occurs from the 1Ls WSe_2_-to-MoS_2_ with an interlayer hexagonal boron nitride (hBN), due to the resonant overlapping of the high-lying excitonic states between the two TMDs, resulting in enhanced HS MoS_2_ PL emission. This type of unconventional ET from the *lower-to-higher* optical bandgap material is not typical in the TMD HSs. With increasing temperature, the ET process becomes weaker due to the increased electron–phonon scattering, destroying the enhanced MoS_2_ emission. Our work provides new insight into the long-distance ET process and its effect on the photocarrier relaxation pathways.

Group-VI semiconducting transition metal dichalcogenides (TMDs) are formed by stacking of strongly bonded two-dimensional (2D) X-M-X layers (M = transition metals such as Mo, W, etc. and X = chalcogens such as S, Se, Te, etc.), which are separated by weak bond interlayer van der Waals forces.^1^ The first mechanical exfoliation of the monolayer (1L) molybdenum disulfide (MoS_2_) film from a bulk crystal in 2010 led us to observe a strong photoluminescence (PL) emission^2,3^ due to the indirect-to-direct bandgap transition from the bulk-to-1L regime.^4,5^ Since then, researchers have been exploring exciting excitonic properties^6−11^ in these 1L TMD materials. In particular, their strong light–matter interactions and high light absorption of up to ∼15% in the solar spectrum^12^ enabled researchers to realize the future prospects of 1L TMD-based optoelectronic device applications.^13^ 2D heterostructures (HSs) made by the vertical stacking of different layered materials have shown promising results for future ultrathin^14−16^ and flexible^17^ optoelectronic device applications. Recent advances in direct and patterned growth of 2D HSs^18,19^ to obtain a clean large-area interface have also pushed the effort to make commercially available TMD-based device applications. However, one of the major challenges in commercializing the promised optoelectronic device applications is the lack of a comprehensive understanding of the competing interlayer processes, such as the interlayer charge transfer (CT) and energy transfer (ET) processes, and their roles in the photocarrier recombination mechanism.

CT and ET are the two main carrier relaxation pathways in the semiconductor HSs. The interlayer CT occurs due to an energy band offset in the HS,^20^ and the interlayer ET process happens when nonradiative energy from the excited donor material gets transferred to the acceptor material accompanied by a fluorescence emission from the acceptor material.^21,22^ ET is observed as a reduction of the donor fluorescence intensity and carrier lifetime followed by an enhancement of the acceptor fluorescence intensity.^22^ The interlayer CT can be stopped by placing a thin layer of dielectric material in between the two semiconductors. Britnell et al.^23^ showed that only four atomic-layer thick hexagonal boron nitride (hBN) is sufficient as a dielectric medium to block the electron tunneling between the two graphene layers. Unlike the CT process, in TMD HSs the long-distance interlayer ET process can survive up to several tens of nm.^24,25^ Separating the materials far apart from each other to stop the ET process is not practical for ultrathin device design. Thus, developing a comprehensive understanding of the long-distance interlayer ET process is an absolute necessity to create practical device applications.

In this work, we study the effect of resonant overlaps between the high-lying excitonic states of 1Ls tungsten diselenide (WSe_2_) and MoS_2_ on the interlayer ET process with a ∼9 nm thick hBN charge-blocking layer. Both these TMD materials have overlapping higher energy B and C (MoS_2_)/D (WSe_2_) absorption features.^26,27^ We show that resonant excitations at the WSe_2_ B and D absorption regions result in MoS_2_ PL enhancement in the HS area. We report that this PL enhancement is due to the interlayer ET process from the WSe_2_-to-MoS_2_ layer. This type of ET process from the *lower-to-higher* optical bandgap material is not typical in the TMD HSs, since ET conventionally happens from the higher-to-lower bandgap 2D materials.^28−33^ In this work, we employ multiple optical spectroscopic techniques at cryogenic temperature (8 K); such as μ-PL, μ-photoluminescence excitation (PLE), and differential reflectance contrast (RC), complemented by the density functional theory (DFT) calculation of spin-resolved band structures to study the ET process. Our work reveals an unconventional interlayer ET process in the TMD HSs. This will significantly contribute to creating a comprehensive understanding of the long-range interlayer ET process and its role to influence the photocarrier radiative recombination processes in these semiconducting HSs.

Figure 1a shows the optical micrograph of the fabricated MoS_2_-hBN-WSe_2_ HS (see the Methods for fabrication details). The inset of Figure 1a shows the schematic illustration of the cross-section of the sample. We introduce a ∼9 nm thick interlayer hBN (see Supplementary Figure S1) to eliminate any effect related to the interlayer CT in our system.^23^ The optical absorption of the TMD materials reflects their single-electron energy band structure. The low temperature RC spectra (see the Methods for details) measured at 8 K show strong overlaps between the B peaks of both materials and of the WSe_2_ D peak with the MoS_2_ C peak (shaded areas in Figure 1b), which agrees well with the previously published reports.^26,27^ In the later sections, we discuss how these strong overlaps help us to observe the reported ET from the *lower-to-higher* bandgap (WSe_2_-to-MoS_2_) material. The HS spectrum (Figure 1b) shows similar RC resonance positions as compared to the individual 1Ls, indicating no major strain-induced effect^34^ in the HS area. A and B excitonic peaks occur due to the excitonic transitions at the K^+^/K^–^ valley in the *k*-space,^2,3^ and higher energy excitonic transitions, such as C and D, are the results of the “band-nesting”^35,36^ in the Brillouin zone. “Band-nested” regions occur due to the identical dispersion in the valence (VB) and conduction (CB) bands over a region in the Brillouin zone due to the strong van Hove singularities. “Band-nesting” regions in TMDs are particularly interesting as the photogenerated electrons and holes propagate with the same but opposite velocities in CB and VB bands, respectively.^35^ For 1L MoS_2_, both the VB maximum and the CB minimum are located at the K^+^/K^–^ point in the Brillouin zone.^2^ In the case of WSe_2_, while the VB maximum is located at the K^+^/K^–^ point, the CB minimum is situated at the Λ point.^37,38^ The “band-nesting” region happens in between the Γ and Λ points.^35,36^Figure 1c shows the DFT calculated electronic band structures (see Supporting Information for details) along the Γ-K^+^ direction in the Brillouin zone. For both the band structures, we match the optical bandgaps with the corresponding PL energies. All types of optical transitions are shown with different colors of arrows (Figure 1c).

**Figure 1:**
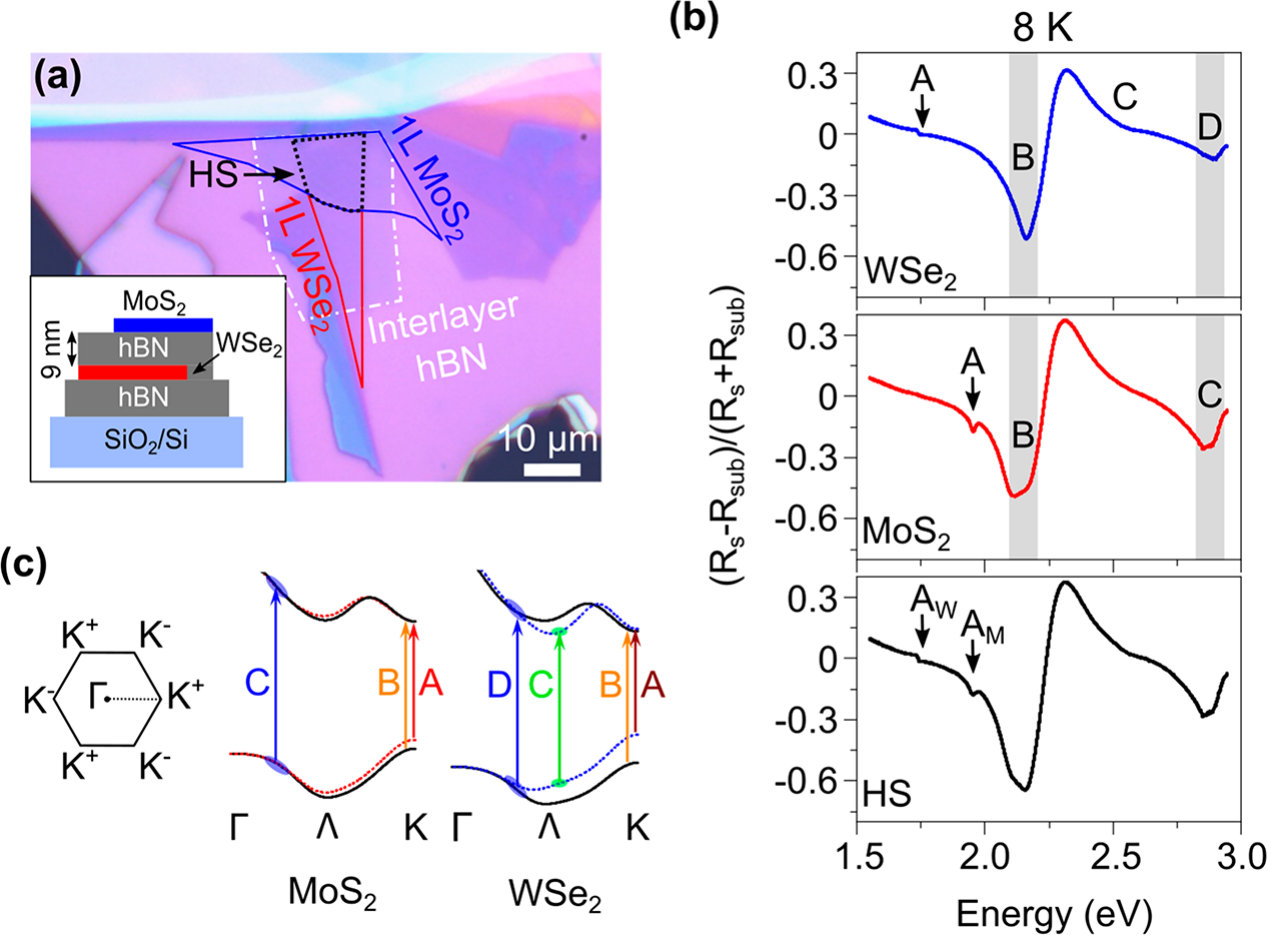
(a) Optical micrograph of the HS. Inset is the schematic illustration of the sample cross-section. The entire MoS_2_ layer is placed on the same hBN thickness. (b) Differential reflectance contrast (RC) spectra from the three areas on the sample taken at 8 K. Shaded areas indicate the higher energy excitonic resonances between MoS_2_ and WSe_2_. HS shows the characteristic lower energy absorptions from both the WSe_2_ (A_W_) and MoS_2_ (A_M_) layers. (c) Single-particle band structure of MoS_2_ and WSe_2_ along the Γ-K direction indicating the different optical transitions. Optical bandgaps were matched with the PL energies. C and D absorption peaks are the results of the “band-nesting” in the Brillouin zone.

PLE maps (see the Methods for the experimental details) taken at 8 K show the emission landscapes of the three individual areas (Figures 2a–2b and S2–S3). In Figure 2a, we saturate the WSe_2_ emission intensity in order to visualize the MoS_2_ emission. After comparing the MoS_2_ emission intensities, we observe a significantly enhanced MoS_2_ PL in the HS area as compared to the 1L region (Figures 2a–2b). The horizontal cuts at the excitation energies of 2.85 and 2.12 eV (black dotted lines in Figures 2a–2b) reveal that the MoS_2_ PL emission in the HS is enhanced by a factor of ∼1.9 and ∼1.7, respectively, as compared to the 1L area (Figures 2c–2d). The PL enhancement factor is defined here as the ratio of PL intensity in the HS area to the 1L area under the same excitation and accumulation conditions. Similarly, the PLE (vertical cut along the 1.92 eV emission energy in Figures 2a–2b) shows an overall increase of the HS MoS_2_ PL emission throughout the entire excitation range as compared to the 1L MoS_2_ region (Figure 2e). It is important to mention that the total optical absorption in the HS area did not change much as compared to each 1L area (Figure 1b). However, the enhancement in the HS PLE (Figure 2e) suggests that the internal PL efficiency of the HS system was increased due to the ET process. We note that the below-bandgap pronounced emission from the MoS_2_ defect states (Figure 2b) is typical for the exfoliated and nonencapsulated samples.^39^ We would also like to mention that in the HS PLE map we also see an enhancement in the WSe_2_ excitonic emission (Figures S2–S3) due to the conventional ET from the MoS_2_-to-WSe_2_ layer. We did not include any discussion related to the enhancement of the WSe_2_ PL in this study, as a similar type of ET has already been reported in a previous work.^28^

**Figure 2:**
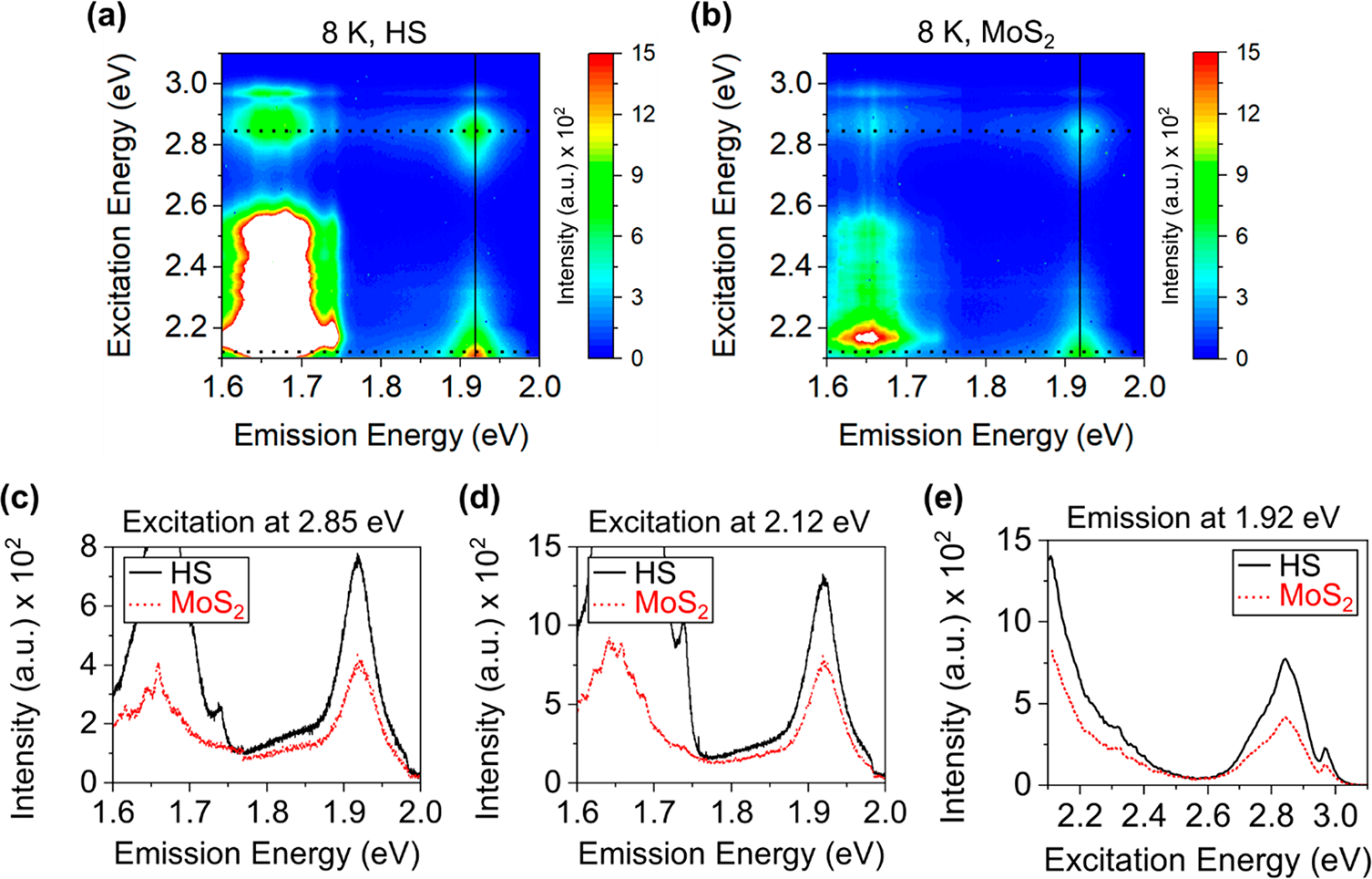
(a–b) PLE maps of the HS and MoS_2_ area with the same intensity range taken at 8 K. The WSe_2_ emission intensity in the HS map is kept saturated to visualize the MoS_2_ emission. MoS_2_ shows a pronounced emission in the HS area. (c–d) (MoS_2_ in) HS and MoS_2_ PL emission intensities at 2.85 and 2.12 eV excitation energies, respectively (along the horizontal dotted lines in parts (a–b). Under both the excited energies, MoS_2_ emissions in the HS are significantly enhanced as compared to the 1L area. (e) Comparison of the HS and MoS_2_ excitation profiles at 1.92 eV emission energy (along the vertical solid lines in part (a–b)). Overall MoS_2_ shows enhanced PLE intensity in the HS area.

In this paragraph, we take into consideration all other possible scenarios in the HS PL enhancement process. We rule out the possibility of the observed PL enhancement in the MoS_2_ emission due to the interference of the backscattering light, because the entire measured MoS_2_ area (including the HS) is placed on the same hBN flake (inset of Figure 1a). 1L WSe_2_ (thickness < 1 nm) in the HS area cannot modulate the interference pattern considering the ∼9 nm interlayer and thick substrate hBNs. We also rule out the possible contribution of ET from the hBN defect states^40^ to the HS MoS_2_ PL enhancement process, as the ET from the same hBN thickness cannot result in more HS PL emission as compared to the 1L MoS_2_ region. In order to check the data reproducibility, we made another HS with a different fabrication protocol and nonidentical hBN thickness and observed an enhanced MoS_2_ PL emission in the HS area (see Supporting Information for details and Figure S4). There is another possibility, that the emitted light from the MoS_2_ layer could be reflected by the encapsulated WSe_2_ at low temperature,^41^ increasing the PL enhancement only at the HS area. To verify this, we made a similar HS on the transparent ultraflat quartz substrate (Figure S5). For this transparent sample we observe shifts in the absorption peaks due to the change in the dielectric environment, which destroys the resonant overlap of the B-excitonic levels between the two materials. As a result, we see a quenching in the HS MoS_2_ PL emission and an increase in the HS WSe_2_ emission, proving that a one-way ET occurred from the MoS_2_-to-WSe_2_ layer.^28^ This result proves that the reflection of the MoS_2_ PL from the encapsulated WSe_2_ layer has no effect here in the reported HS PL enhancement process. We conclude that the MoS_2_ PL enhancement in the HS area is a result of an interlayer ET process from the WSe_2_ layer.

Strong overlaps between the higher energy absorptions in both the investigated materials (Figure 1b) help us to study the effect of the interlayer ET process under those “resonant” excitation conditions. The PL intensity map taken at 8 K under the excitation of 2.12 eV (B resonances overlap region) shows an overall enhanced MoS_2_ emission in the HS area (Figure 3a). Similarly, an excitation at 2.85 eV energy (WSe_2_ D and MoS_2_ C peaks overlap region) shows an increased MoS_2_ PL emission throughout the HS area (Figure 3b), thus proving that at both the excitation energies an efficient ET happened from the WSe_2_-to-MoS_2_ layer as discussed in the later section. The PL intensity maps (Figures 3a–3b) also show that the observed enhancement of the MoS_2_ PL emission in the HS area is not a localized phenomenon. We note that although there is some nonuniformity in the HS PL intensity due to the typical inhomogeneous nature of the exfoliated samples, but the HS PL emission is always higher than the 1L MoS_2_ area.

**Figure 3:**
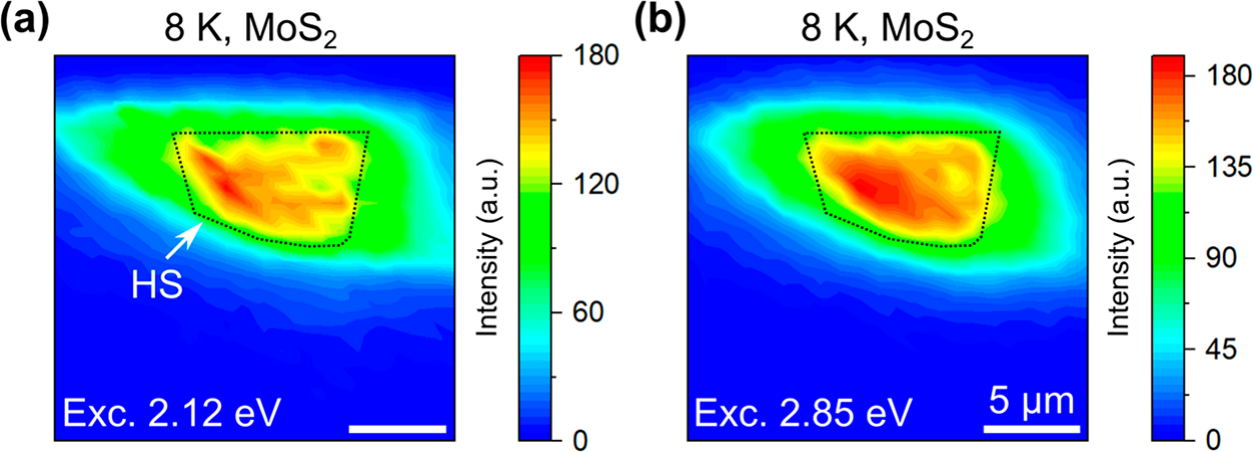
MoS_2_ photoluminescence (PL) intensity maps at 8 K under (a) 2.12 and (b) 2.85 eV excitation energy. MoS_2_ emission in the HS area shows an overall increased PL emission as compared to the 1L region. The scale bars represent 5 μm length.

In order to study the effect of increasing temperature in our experiments, we performed PLE maps at 25, 100, and 200 K (Figure 4 and Figure S6). At 25 K, MoS_2_ emissions in the HS area under both the excitation energies at ∼2.83 and 2.2 eV show a similar enhancement factor of ∼1.6 (Figure S7). These values are a slight reduction from the 8 K data. The PLE also shows a similar overall enhancement in the MoS_2_ HS emission at 25 K (Figure 4c). Upon further increasing the temperature at 100 and 200 K, we observe a complete vanishing of the MoS_2_ PL enhancement in the HS (Figures 4d–4f). A slight quenching of the HS MoS_2_ PLE at 100 K (Figure 4f) could be due to the conventional type-II HS ET^28^ from the higher-to-lower bandgap material (MoS_2_-to-WSe_2_).

**Figure 4:**
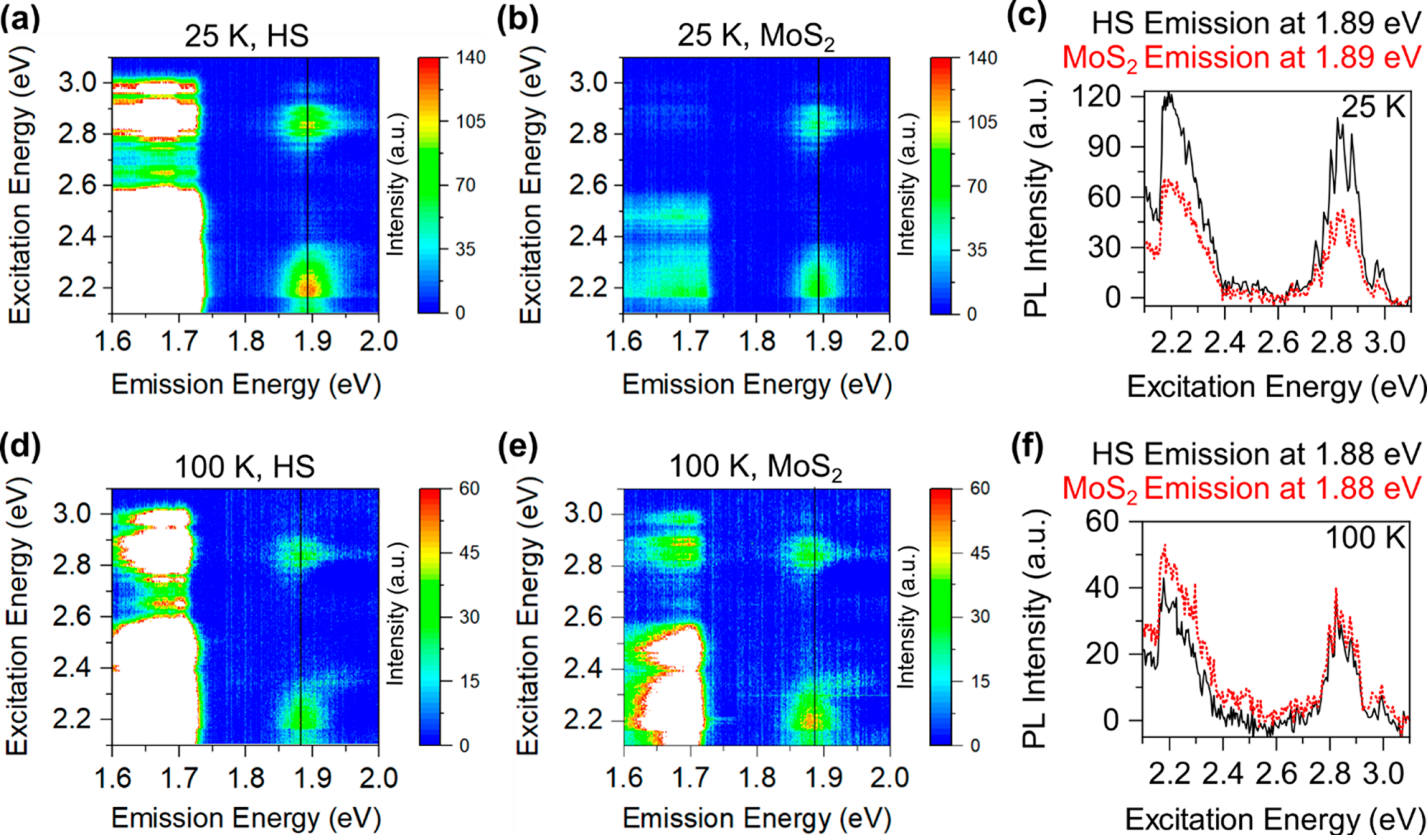
(a–b) HS and MoS_2_ PLE maps at 25 K. MoS_2_ PL emission in the HS area shows an enhancement as compared to the 1L area. (c) HS and MoS_2_ PLE comparison along the vertical lines in parts (a–b). HS shows a slightly reduced MoS_2_ PLE enhancement as compared to the 8 K map. (d–e) HS and MoS_2_ PLE maps taken at 100 K. (f) Similar HS and MoS_2_ PLE comparison at 100 K. MoS_2_ in the HS area does not show any intensity enhancement at 100 K as compared to the 1L area. In all the HS maps, WSe_2_ emission intensities are kept saturated to visualize the MoS_2_ emission.

For MoS_2_ and WSe_2_, the schematics of the A and B transitions based on the VB and CB splitting are shown in Figure 5a. In these TMD monolayers, VB (VB1 and VB2) and CB (CB1 and CB2) spin splitting occurs due to the spin–orbit coupling and lack of inversion symmetry,^10,42^ allowing possible absorptions based on the optical selection rule.^43,44^ The corresponding PL emission, which comes from the direct radiative recombination at the optical bandgap, strongly depends on the spin-state of the CB (CB1 or CB2) electron and the VB (VB1 or VB2) hole at the K^+^/K^–^ point. Based on the allowed electron recombination from the CB1 or CB2 to the hole situated at the top of the VB (VB2), the materials are divided into two categories: “bright” or “dark”, respectively.^10^ The calculated momentum-space energy landscape for the allowed optical transitions from VB2-to-CB1 and VB1-to-CB2 in the MoS_2_ layer shows a smaller separation of ∼150 meV at the K^+^/K^–^ point due to the spin splitting (Figures 5b–5c and S8a), which matches well with the previous results.^45,46^ WSe_2_ shows a comparatively larger separation of ∼500 meV at the K^+^/K^–^ point^47,48^ for the VB2-to-CB2 and VB1-to-CB1 transitions (Figures 5d–5e and S8b). The spin- and momentum-resolved energy landscapes (Figures 5b–5e) help us to visualize the optical transitions corresponding to the PLE energies and eventually deduce the clear picture of the possible photocarriers’ relaxation pathways.

**Figure 5:**
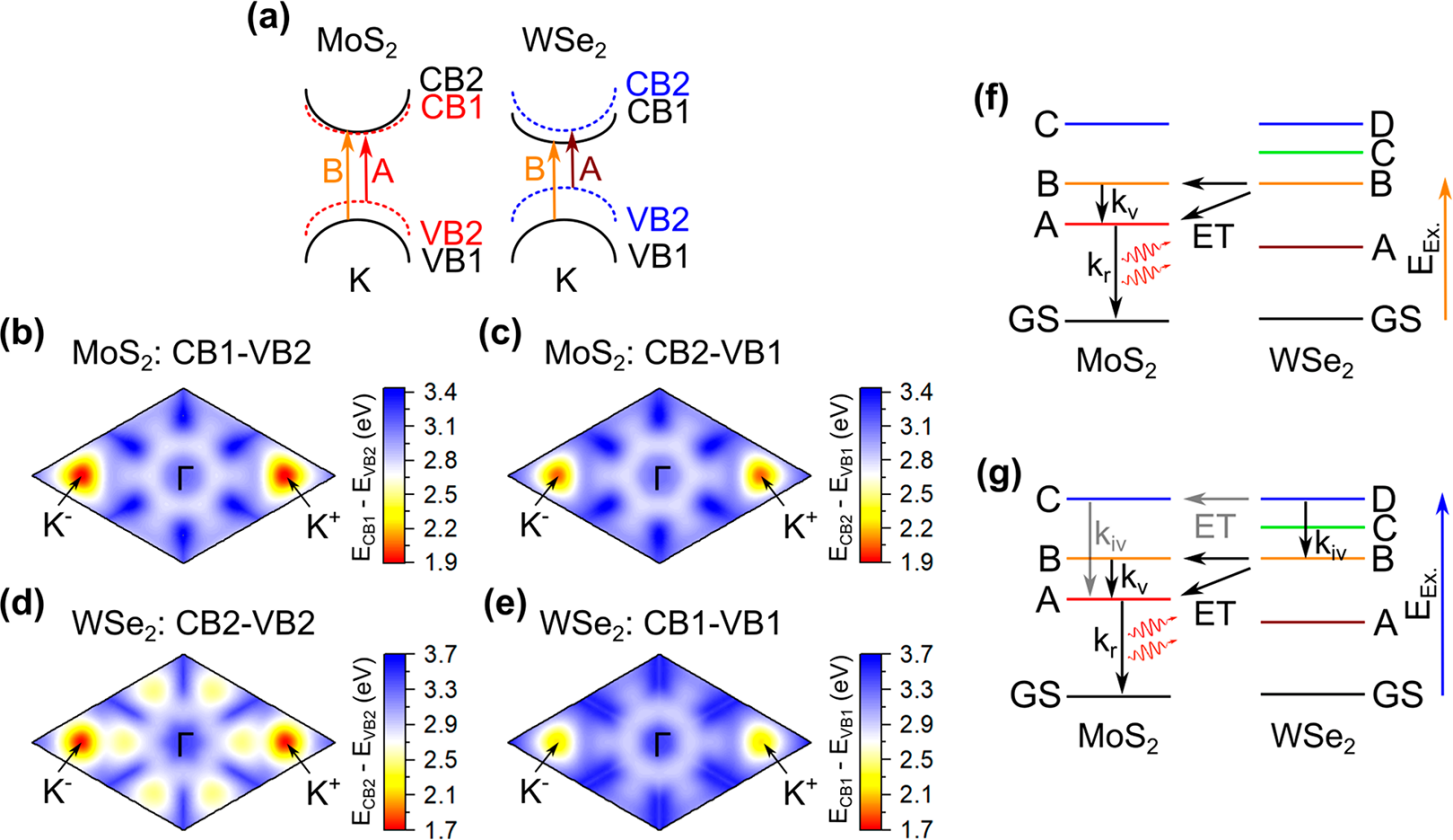
(a) Schematic illustration of the valence band (VB) and conduction band (CB) splitting at the K valley in MoS_2_ and WSe_2_, respectively. (b–c) Calculated MoS_2_ optical transitions along the K^–^-Γ-K^+^ direction from VB2 to CB1 and VB1 to CB2 (as shown in part (a)), respectively. (d–e) Similar calculated WSe_2_ momentum-space energy landscape along the K^–^-Γ-K^+^ direction from VB2 to CB2 and VB1 to CB1 (as shown in part (a)), respectively. (f–g) Schematic illustration of the photocarrier relaxation pathways from the higher energy levels to the ground state (GS) in MoS_2_ due to the energy transfer (ET) from WSe_2_ after resonant excitation at the (WSe_2_) B and D excitonic levels, respectively. Different types of transition are shown in the MoS_2_ layer; such as intravalley scattering (*k*_iv_), intervalley transition (*k*_v_), and radiative recombination (*k*_r_).

Optical excitation at the “band-nested” region (MoS_2_ C and WSe_2_ D peaks) excites electrons in the valley in between the Γ–Λ point in the MoS_2_ CB and around the Λ valley in the WSe_2_ CB. These excited photocarriers (electron and hole) instantly relax to their immediate band extreme points: the Λ valley for the electrons and the Γ hill for the holes.^27^ These carriers then further transfer to the band extrema *via* the extremely fast (<500 fs) intravalley scattering (*k*_iv_).^49−51^ In our HS, to describe the PL intensity map under the 2.12 eV excitation (Figure 3a), the only possible mechanism is shown as a schematic illustration in Figure 5f. Upon excitation with the 2.12 eV photons, photoexcited carriers are generated at the WSe_2_ B excitonic level. Due to the resonant overlap with the MoS_2_ B level (Figure 1b), the WSe_2_ B excitonic energy immediately transfers to the MoS_2_ B and A bands, resulting in more carriers in the MoS_2_ layer. The extra carriers at the MoS_2_ B level transfer to the subsequent band extremum *via* an intervalley transition (*k*_v_, i.e., B_K+/K-_ to A_K-/K+_ transitions), followed by a radiative recombination process (*k*_r_) to the ground state (GS). Thus, we obtain an enhanced MoS_2_ PL emissions in the HS area with an excitation of 2.12 eV (Figure 3a). However, at an excitation energy of 2.85 eV (MoS_2_ C and WSe_2_ D peaks overlap region, Figure 1b), two possible ET channels can play a crucial role. First, ET from the WSe_2_ D level can directly generate more carriers at the MoS_2_ C level due to the resonant overlapping. These extra carriers radiatively recombine at the band extremum *via* intravalley transition (*k*_iv_) and give rise to more MoS_2_ PL emissions in the HS area, as shown in the schematic of Figure 5g (gray colored ET process). Another possibility is that upon the 2.85 eV excitation carriers generated at the WSe_2_ D level scatter to the WSe_2_ B level *via* the analogous *k*_iv_ process and then transfer to the MoS_2_ B and A levels *via* the ET process. Finally, it increases the MoS_2_ PL emission similar to the 2.12 eV excitation process (black colored ET process in Figure 5g). Interestingly, an excitation at the WSe_2_ C absorption peak (2.56 eV) does not result in any MoS_2_ PL emission (Figure S9), indicating that interlayer coupling between the suitable levels was not possible at this excitation due to the immediate photoexcited carrier transfer to the WSe_2_ A level. Hence, no enhancement in the MoS_2_ HS PL emission due to the ET process is also apparent.

Our model to describe the enhanced MoS_2_ PL emission from the HS area also supports the temperature-dependent data. Photocarriers go through a series of phonon scattering before relaxing to the ground state. At low temperature, electron–phonon scattering dominates.^52^ With the increasing temperature, other types of scattering processes, such as anharmonic phonon–phonon scattering and phonon structure scattering,^53^ start to dominate. Thus, with the increasing temperature, the intravalley transition becomes weaker due to the multiple-phonon scattering and eventually a minor fraction of the photocarriers generated at the “band-nested” region can be transferred to the K^+^/K^–^ point for radiative recombination. Furthermore, the thermal activation should make the “hot” carrier transfer to the band extremum extremely faster (<100 fs),^54^ preventing the coupling between the materials’ corresponding energy levels. These eventually result in a complete disappearance of the MoS_2_ PL enhancement in the HS area at higher temperatures (100 and 200 K).

Considering the temperature-dependent data, we can conclude that at higher excitation energy (∼2.85 eV) the ET process *via* the WSe_2_ B level to the MoS_2_ B and A levels dominates (black colored ET process in Figure 5g) in our experiment. Otherwise, with increasing the temperature we should observe an enhanced MoS_2_ HS PL emission. At cryogenic temperature, the fast intravalley scattering (*k*_iv_) in TMDs occurs at the ∼100–500 fs time scale,^49−51,54^ whereas intervalley transitions (*k*_v_) occur at a longer time scale of a few ps range.^55,56^ Our study suggests that the reported ET happened at a time scale faster than the intervalley transition and slower than the intravalley transition. Otherwise, the ET from the lower optical bandgap WSe_2_ cannot excite more carriers in the higher bandgap MoS_2_, resulting in an enhanced HS MoS_2_ PL emission. Finding the “true” ET time scale in our experiment will require an ultrafast study, which is beyond the scope of this work. We would like to point out that the effect of trions participating in the ET process cannot be excluded, as the binding energies between the trions and excitons at low temperature are only in the order of a few tens of meV in WSe_2_^57^ and MoS_2_.^58^ Resolving the MoS_2_ excitons–trions contribution is beyond the resolution limit of our instrumental setup. However, this does not change the overall picture of our work. It is also important to mention that with the increasing temperature the effect of band renormalization in the ET process to alter the radiative recombination pathway of the photocarriers cannot be ignored. A thorough investigation of the band renormalization effect in the ET process is required in the future work.

In conclusion, our study shows that strong light–matter interaction in the 1L MoS_2_ and WSe_2_ “band-nested” region allows us to observe an unusual ET process from the *lower-to-higher* bandgap (WSe_2_-to-MoS_2_) material. The excitation-dependent PL intensity maps prove that the reported HS MoS_2_ PL enhancement is not a localized phenomenon due to the material’s local property change; the entire HS area shows this enhanced PL emission. Finally, the temperature-dependent study proves that with the increasing temperature due to the growing electron–phonon scattering, the carriers’ transfer to the band extremum becomes faster, preventing ET from the WSe_2_ (smaller gap) to the MoS_2_ (larger gap) layer. Our findings provide an important insight into the interlayer ET process in these layered materials and will help to build a comprehensive understanding about the competing interlayer processes for developing future TMD-based optoelectronic device applications.

## Methods

### HS Fabrication

We fabricated three HSs using two fabrication protocols. HSs in Figures 1a and S5, were fabricated using the PDMS-based dry transfer technique at the University of Warsaw. The bottom hBN layer was directly cleaved on the SiO_2_/Si substrate. MoS_2_-hBN-WSe_2_ layers were exfoliated onto the Gel-Pak (PDMS) films and were stacked layer-by-layer (in reverse order) onto each other using a home-built semiautomatic transfer stage. The other HS in Figure S4 was partially fabricated using a robotic fabrication system (QPress) at the Brookhaven National Laboratory (the details in Supporting Information). MoS_2_, WSe_2_, and hBN bulk crystals for exfoliation were obtained from the Graphene Supermarket, HQ Graphene, and National Institute for Materials Science, respectively.

### Characterization

We used a Bruker Dimension Icon with NanoScope 6 controller in ‘ScanAsyst’ (peak force tapping) mode to obtain the high-resolution AFM images.

The differential RC measurements were performed using a supercontinuum light source (without a monochromator) focused by a Nikon L Plan 100× (N.A. 0.7) objective and directed into a spectrometer. Samples were loaded in a cryostat and cooled with a continuous flow of liquid helium (LHe). The differential reflectance is defined by (*R*_s_ – *R*_sub_)/(*R*_s_ + *R*_sub_), where *R*_s_ is the reflected light intensity from the TMD sample areas and *R*_sub_ is that from the hBN/Si substrate.

We performed the μ-PL/PLE experiments by using a supercontinuum light source coupled with a monochromator as an excitation source. The incident light was focused using a Mitutoyo M Plan 50× (N.A. 0.75) objective. The excitation power was constant throughout the measurements, and the average power on the sample was kept at ∼50 μW (spot diameter ∼ 1 μm) to avoid any high-power-induced nonlinear effects from the sample. For the PLE experiment sample was loaded in a LHe cryostat to reach the minimum temperature of ∼5 K during the experiments.

## Data Availability

All the data necessary to conclude the results are presented in the manuscript and Supporting Information. The technical details of the theoretical calculations are available from the corresponding authors upon reasonable request.

## Supporting Information Available

Details of the theoretical calculations, AFM height profile, extended PLE maps and spectra of different HSs, RC spectra of HS sample on transparent substrate, PL intensity map, and spin-resolved DFT calculated energy landscape (PDF)
