# Supplementary material for: Excitation-Dependent High-Lying Excitonic Exchange via Interlayer Energy Transfer from Lower-to-Higher Bandgap 2D Material
Source: Nano Lett. 2023 Jun 8;23(12):5617–24. doi: 10.1021/acs.nanolett.3c01127 (PMC10311602; doi:10.1021/acs.nanolett.3c01127)
Supplement: Supplementary file 1 — nl3c01127_si_001.pdf [file nl3c01127_si_001.pdf]

SUPPORTING INFORMATION

Excitation-Dependent High-Lying Excitonic Exchange *via* Interlayer Energy Transfer from *Lower-to-Higher* Bandgap 2D Material

Arka Karmakar[1*], Tomasz Kazimierczuk[1], Igor Antoniazzi[1], Mateusz Raczyński[1], Suji Park[2], Houk Jang[2], Takashi Taniguchi[3], Kenji Watanabe[4], Adam Babiński[1], Abdullah Al-Mahboob[2ɫ], Maciej R. Molas[1#]

[1] Division of Solid State Physics, Institute of Experimental Physics, Faculty of Physics, University of Warsaw, Pasteura 5, 02-093 Warsaw, Poland

[2] Center for Functional Nanomaterials, Brookhaven National Laboratory, Upton, NY 11973, USA

[3] International Center for Materials Nanoarchitectonics, National Institute for Materials Science, 1-1 Namiki, Tsukuba, Ibaraki 305-0044, Japan

[4] Research Center for Functional Materials, National Institute for Materials Science, 1-1 Namiki, Tsukuba, Ibaraki 305-0044, Japan

[*] arka.karmakar@fuw.edu.pl

[ɫ] aalmahboo@bnl.gov

[#] maciej.molas@fuw.edu.pl

Details of the theoretical calculations:

We computed the ground state band structure of 1Ls MoS_2 and WSe_2 employing the density functional theory (DFT) calculations using the Materials Studio CASTEP (CAmbridge Serial Total Energy Package) version 2021 HF1, *ab initio* Total Energy Program (first principles methods using CASTEP).[1] Prior to the band structure calculation, we performed the geometry optimization (GO) for the bulk crystal structure using DFT-D (GGA + dispersion correction) method - Perdew-Bruke-Ernzerhof (PBE) GGA functional[2] along with the dispersion correction (van der Waals correction accounted employing the dispersion correction for DFT) by Tkatchenko-Scheffler (TS) method,[3] which was performed using the DFT Semi-Empirical Dispersion Interaction Correction (DFT-SEDC) module.[4] We obtained the electron relativistic correction using the DSPP (DFT-Semicore Pseudopotential).[5] During the GO of the bulk structure, symmetry constrained was imposed considering the International Table #194 (hexagonal, symmetry group $P6_3/mmc$, crystal class 6/mmm) for the bulk MoS_2 and WSe_2. Following the bulk geometry optimization, crystal was cleaved parallel to the layer (c* terminated) and then a vacuum slab > 20 Å was added along the c* to make the 1L TMD structures. Final GO for the atomic arrangement within the 1L and the in-plane lattice parameters were further optimized constraining the 2D lattice symmetry employing the identical GGA functional and dispersion correction as above but also including the spin-orbit coupling in the total energy calculations. In order to include the spin-orbit coupling, norm-conserving potentials in CASTEP were generated using the kinetic energy optimization scheme developed by Lin *et al.*[6] The spin orbit coupling was included using the j-dependent pseudopotentials developed for CASTEP based on the work by *ref.*[7] Following the final step of GO, band structure calculation was performed considering the ultra-fine k-spacing (k-spacing in single point energy calculation corresponding to 50x50x1 supercell or better and spectral k-spacing of 0.0005Å-1).

After computation of the electronic band structure in CASTEP, scissors have applied to the band structure plot to match with the bandgap obtained from the PL spectroscopy measurements.

AFM data of the interlayer hBN thickness of the main MoS_2-hBN-WSe_2 sample (Figure S1):

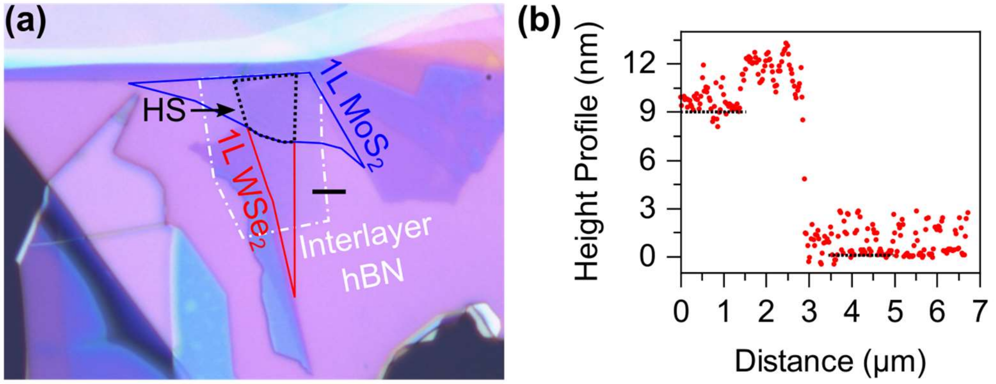

Figure S1: (a) Optical micrograph of the HS. Solid black line indicates the line region of the AFM height profile. (b) AFM height profile of the interlayer hBN shows the thickness of ~9 nm.

Full PLE map of the HS and 1L WSe_2 region from the main MoS_2-hBN-WSe_2 sample (Figure S2-S3):

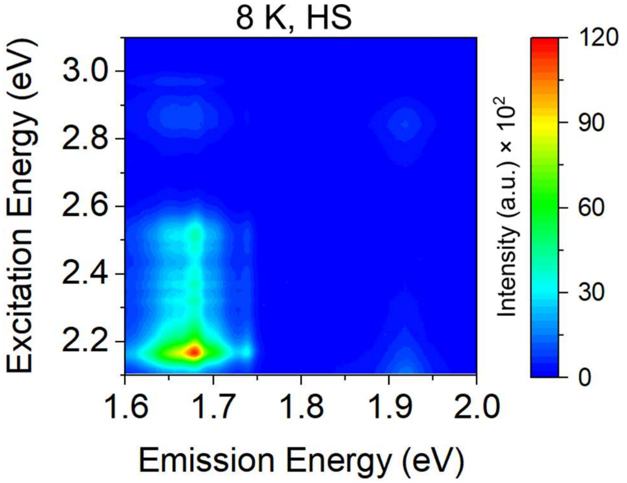

Figure S2: HS PLE map taken at 8K to visualize the WSe_2 emission.

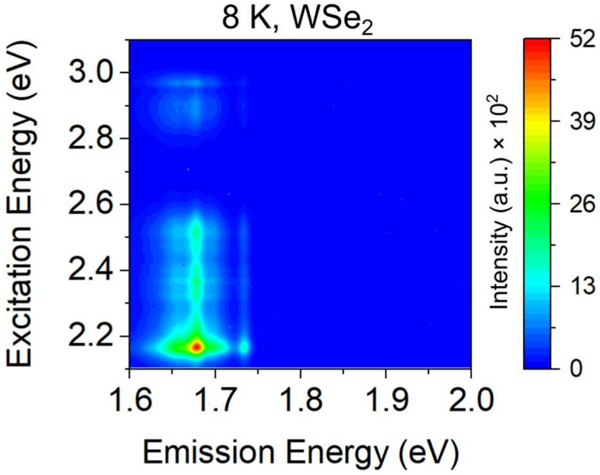

Figure S3: WSe_2 PLE map taken at 8K. WSe2 excitonic intensity is reduced as compared to the HS area (Figure S2).

HS fabrication using Quantum material press (QPress) facility at Brookhaven National Laboratory (Figure S4):

Each layer (except MoS_2) was exfoliated on SiO_2/Si substrates by a roll-to-roll exfoliation machine (or R2R exfoliator) in QPress. Exfoliation conditions were controlled following the recipes developed and provided by the QPress facility. We transferred the exfoliated flaked by a dry transfer method using a stamp consisting of a thin polycarbonate (PC) film onto a polydimethylsiloxane (PDMS) dome mounted on a glass slide. Unlike PDMS-based transfer method (bottom-up), the interlayer hBN was picked up by the PC/PDMS stamp first and then WSe_2 and bottom hBN was picked up sequentially (top-down). When picking-up, the substrate was heated up to ~150 °C. The (interlayer) hBN-WSe_2-(bottom) hBN structure stacked under the PC/PDMS stamp was released onto the final SiO_2/Si substrate by melting the PC layer at ~180 °C. PC residues were rinsed by chloroform, acetone and isopropanol. We soft baked the sample before transferring the top MoS_2 layer using the PDMS-based dry transfer technique as mentioned in the main text. We did not complete the entire HS using the PC/PDMS stamp to protect the top MoS_2 layer from chemical doping during the chloroform, acetone and isopropanol treatment.

Due to an instrumental limitation the PLE measurements were performed in the range of ~2.12-2.5 eV excitation energies with an average power ~40 μW. Figure S4 shows the optical image and PLE maps of the sample.

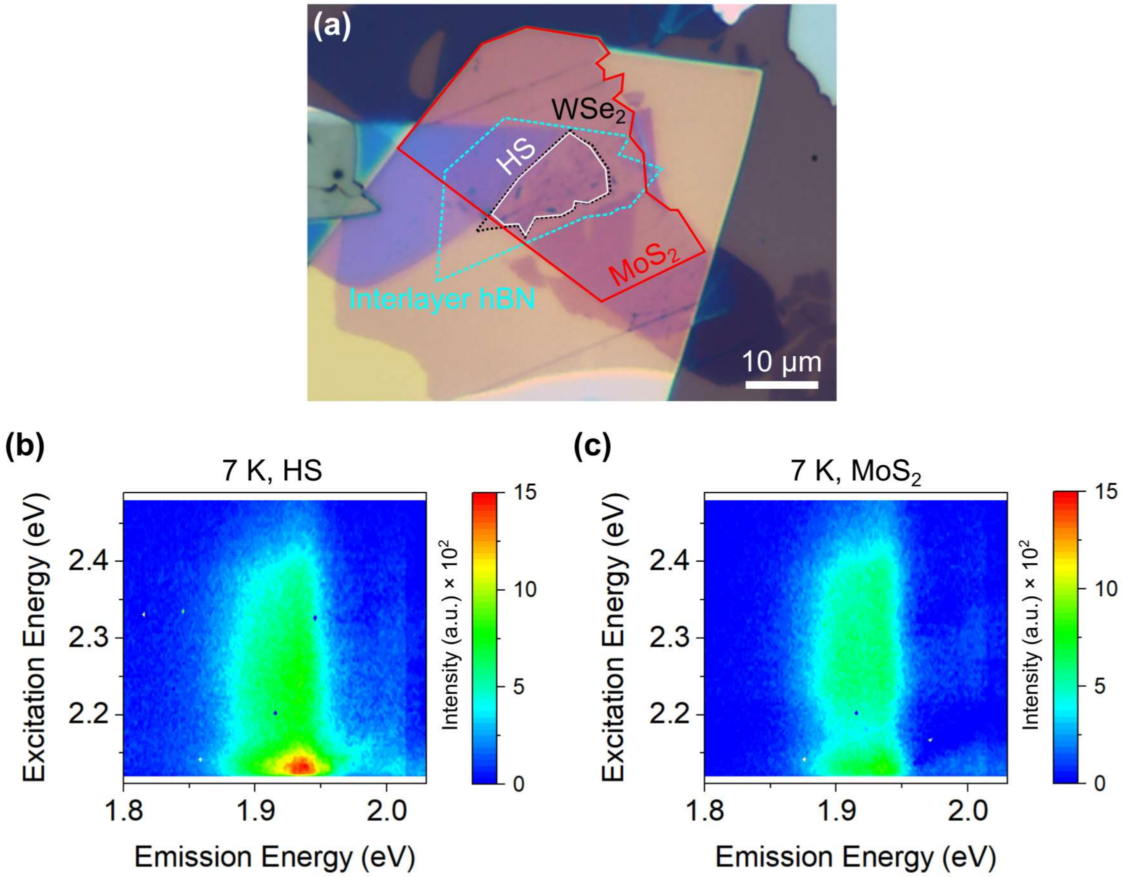

Figure S4: (a) Optical micrograph of the MoS_2-hBN-WSe_2 HS. (b)-(c) PLE maps of the HS and MoS_2 taken at 7 K, respectively. MoS_2 PL emission shows an increased intensity in the HS area. Both the plots have the same intensity range.

MoS_2-hBN-WSe_2 HS on transparent quartz substrate (Figure S5):

This HS on the ultraflat transparent quartz substrate was fabricated using the PDMS-based transfer technique as described in the method section of the manuscript. RC spectra taken at 6 K show the mismatch between the B excitonic level of the two materials due to the change in the dielectric environment. WSe_2 and MoS_2, A and B absorption peaks are marked with the dotted lines. This mismatch in the B excitonic level results in an one way ET process from the MoS_2-to-WSe_2 layer, as observed by a previous report.[8] As a result, MoS_2 PL emission decreases in the HS area (pointed by black arrows), but the WSe_2 emission is enhanced in the HS region (pointed by white arrows).

The PLE measurements were performed in the range of ~2.12-2.5 eV excitation energies with an average power ~40 μW. Figure S5 shows the optical image, RC spectra and PLE maps of the sample.

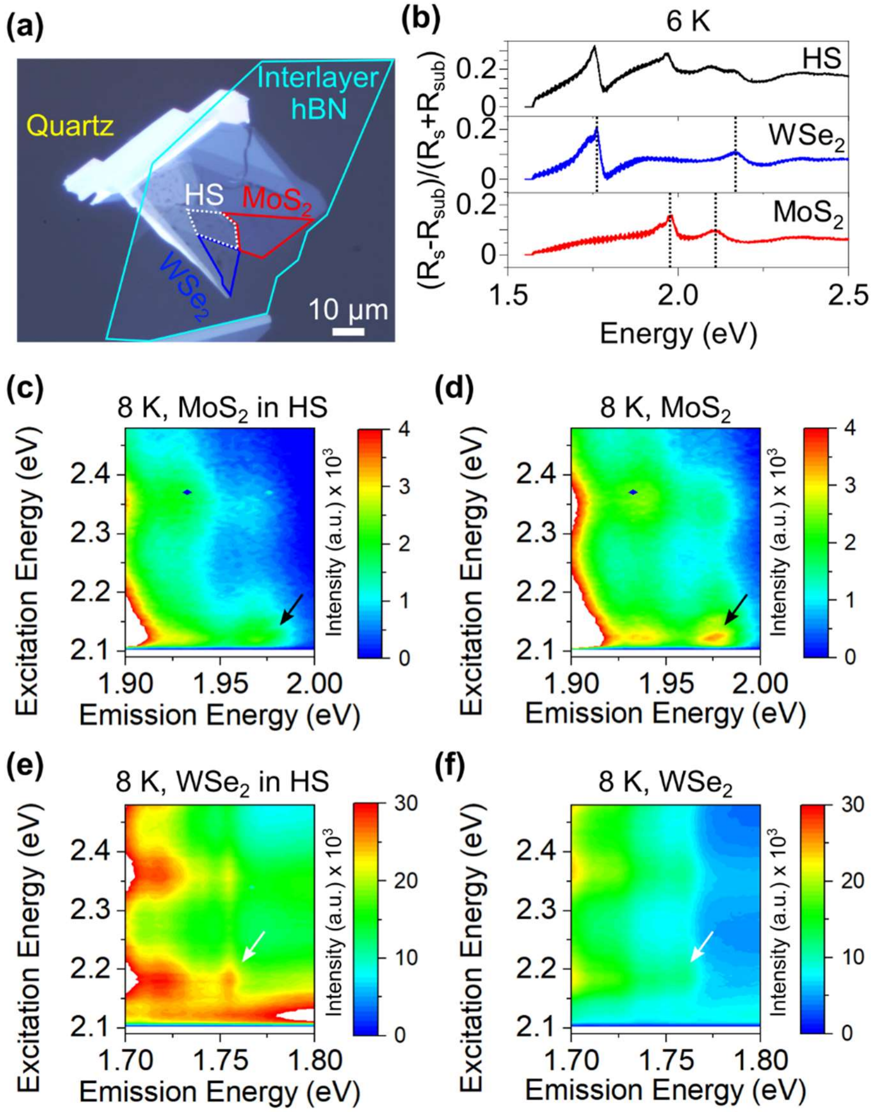

Figure S5: (a) Optical micrograph of the MoS_2-hBN-WSe_2 HS on transparent quartz substrate. (b) RC spectra of the sample from the three different regions taken at 6 K. B excitonic resonance between the two material breaks due to the change in dielectric environment. (c)-(d) PLE maps of the MoS_2 in HS and 1L MoS_2 taken at 8 K, respectively. MoS_2 PL emission does not show an increased intensity in the HS area. Both the plots have the same intensity range. Black arrows indicate the MoS_2 excitonic emission. (e)-(f) PLE maps of the WSe_2 in HS and 1L WSe_2 taken at 8 K, respectively. WSe_2 emission in the HS area shows an enhanced intensity, proving that only one-way ET happened from the MoS_2-to-WSe_2 layer. Both the maps have the same intensity range. White arrows indicate the WSe_2 excitonic emission.

PLE maps of the HS and MoS_2 area taken at 200 k from the main sample (Figure S6):

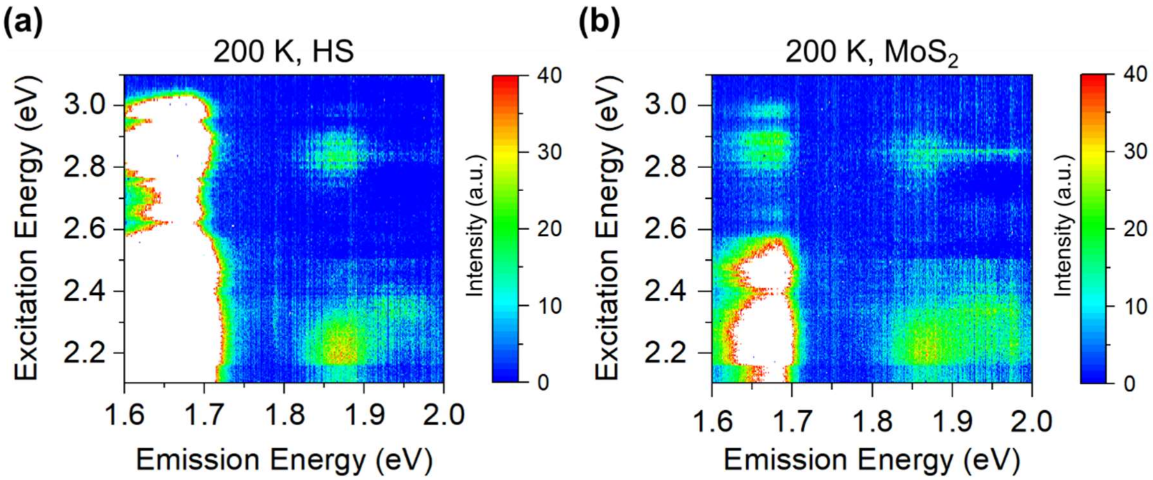

Figure S6: (a)-(b) PLE maps of the HS and MoS_2 at 200 K, respectively. MoS_2 PL emission does not increase in the HS area. WSe_2 emission in the HS data is saturated to visualize the MoS_2 emission. Both the plots have the same intensity range.

HS and MoS_2 PL intensity comparisons at WSe_2 D and B excitations, respectively (Figure S7):

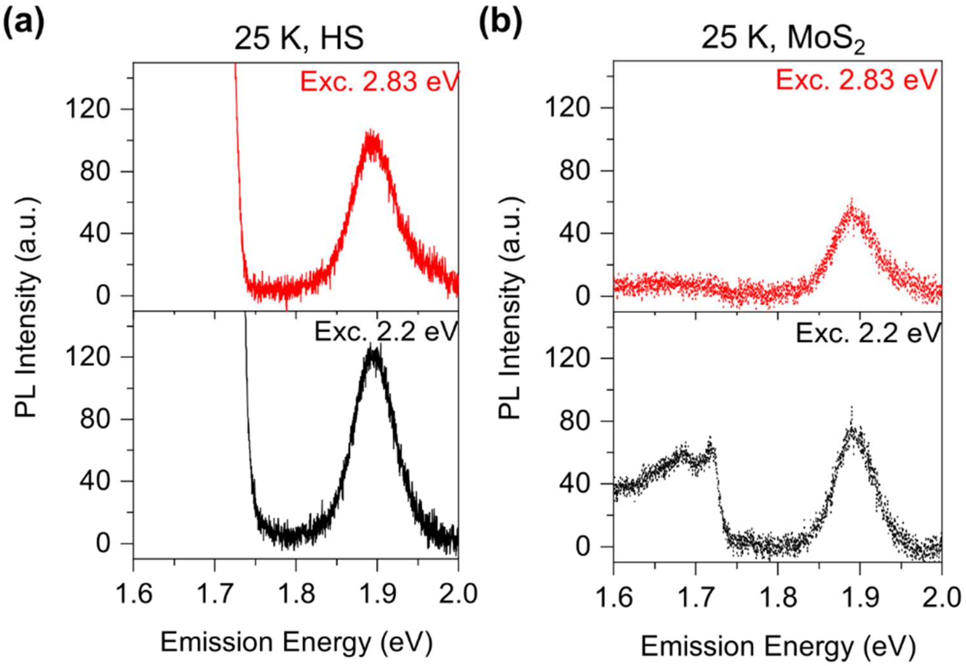

Figure S7: (a) Top and bottom panel shows PL emission of the MoS_2 in the HS area under excitation at 2.83 eV and 2.2 eV, respectively. (b) PL emission profile from the 1L MoS_2 area under same excitation conditions. MoS_2 PL emission in the HS area shows similar enhancement factor of ~ 1.6 at both excitation energies.

DFT calculated MoS_2 and WSe_2 spin-resolved energy landscape along the Γ-K direction (Figure S8):

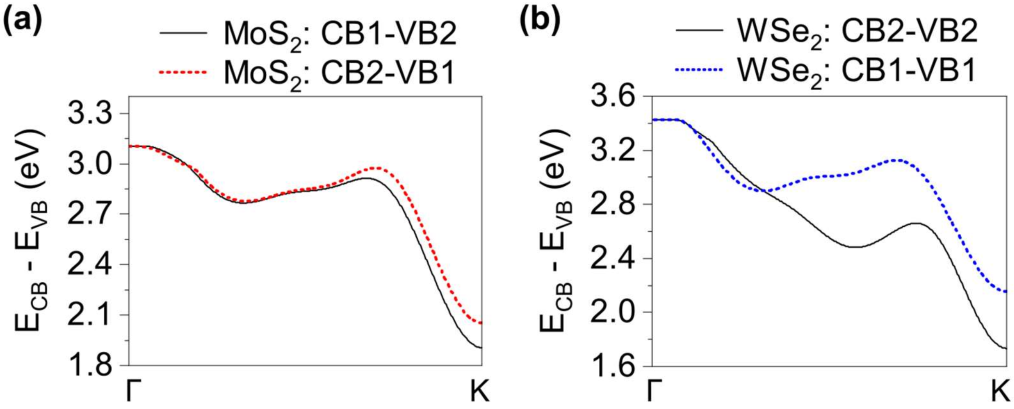

Figure S8: Calculated spin-resolved momentum-space optical absorption energy landscape of 1L (a) MoS_2 and (b) WSe_2 along the Γ-K direction in the Brillouin zone.

PL intensity map of the MoS_2 emission at WSe_2 C excitation (Figure S9):

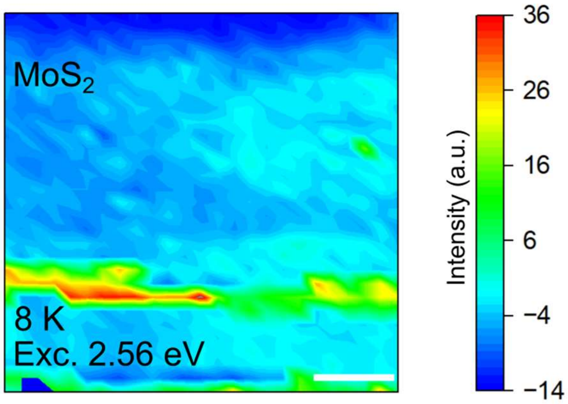

Figure S9: PL Intensity maps at the resonant WSe_2 C excitation (~ 2.56 eV). MoS_2 does not show any PL emission at this excitation energy. Only system noise was detected in this condition. Scale bar represents 5 μm length.

References:

(1) Clark, S. J.; Segall, M. D.; Pickard, C. J.; Hasnip, P. J.; Probert, M. I. J.; Refson, K.; Payne, M. C. First Principles Methods Using CASTEP. *Zeitschrift für Kristallographie - Crystalline Materials* **2005**, *220* (5–6), 567–570. https://doi.org/10.1524/zkri.220.5.567.65075.

(2) Perdew, J. P.; Burke, K.; Ernzerhof, M. Generalized Gradient Approximation Made Simple. *Phys. Rev. Lett.* **1996**, *77* (18), 3865–3868. https://doi.org/10.1103/PhysRevLett.77.3865.

(3) Tkatchenko, A.; Scheffler, M. Accurate Molecular Van Der Waals Interactions from Ground-State Electron Density and Free-Atom Reference Data. *Phys. Rev. Lett.* **2009**, *102* (7), 073005. https://doi.org/10.1103/PhysRevLett.102.073005.

(4) McNellis, E. R.; Meyer, J.; Reuter, K. Azobenzene at Coinage Metal Surfaces: Role of Dispersive van Der Waals Interactions. *Phys. Rev. B* **2009**, *80* (20), 205414. https://doi.org/10.1103/PhysRevB.80.205414.

(5) Delley, B. Hardness Conserving Semilocal Pseudopotentials. *Phys. Rev. B* **2002**, *66* (15), 155125. https://doi.org/10.1103/PhysRevB.66.155125.

(6) Lin, J. S.; Qteish, A.; Payne, M. C.; Heine, V. Optimized and Transferable Nonlocal Separable Ab Initio Pseudopotentials. *Phys. Rev. B* **1993**, *47* (8), 4174–4180. https://doi.org/10.1103/PhysRevB.47.4174.

(7) Corso, A. D.; Conte, A. M. Spin-Orbit Coupling with Ultrasoft Pseudopotentials: Application to Au and Pt. *Phys. Rev. B* **2005**, *71* (11), 115106. https://doi.org/10.1103/PhysRevB.71.115106.

(8) Kozawa, D.; Carvalho, A.; Verzhbitskiy, I.; Giustiniano, F.; Miyauchi, Y.; Mouri, S.; Castro Neto, A. H.; Matsuda, K.; Eda, G. Evidence for Fast Interlayer Energy Transfer in MoSe2/WS2 Heterostructures. *Nano Lett.* **2016**, *16* (7), 4087–4093. https://doi.org/10.1021/acs.nanolett.6b00801.
